# The Heart Is a Smart Pump: Mechanotransduction Mechanisms of the Frank-Starling Law and the Anrep Effect

**DOI:** 10.1146/annurev-physiol-022724-104846

**Published:** 2025-02-03

**Authors:** Ye Chen-Izu, Tamas Banyasz, John A. Shaw, Leighton T. Izu

**Affiliations:** 1 Department of Pharmacology, School of Medicine, University of California, Davis, California, USA; 2 Department of Biomedical Engineering, University of California, Davis, California, USA; 3 Department of Internal Medicine, Division of Cardiovascular Medicine, University of California, Davis, California, USA; 4 Department of Physiology, University of Debrecen, Debrecen, Hungary; 5 Department of Aerospace Engineering, University of Michigan, Ann Arbor, Michigan, USA

**Keywords:** heart, autoregulation, cardiomyocyte, mechano-chemo-transduction, Ca^2+^ signaling, excitation-contraction coupling

## Abstract

The Frank-Starling law and Anrep effect describe two intrinsic mechanisms that regulate contraction force in the heart. Based on recent advancements and the historical literature, we propose new perspectives and address several critical issues in this review. (*a*) The Frank-Starling mechanism and Anrep effect are dynamically linked and act synergistically. (*b*) An open question is how cardiomyocytes sense mechanical load and transduce to biochemical signals (called mechano-chemo-transduction or MCT) to regulate contraction in response to load changes. (*c*) One research focus is to identify various mechanosensors and decipher their downstream MCT pathways. (*d*) Innovative experimental techniques engage different mechanosensors that detect different local strain and stress in the cell architecture. (*e*) Closed-loop MCT feedback in the dynamic excitation-Ca^2+^ signaling-contraction system enables autoregulation of contraction in response to physiological load changes. (*f*) However, pathological overload such as volume and pressure overload lead to excessive MCT-Ca^2+^ gain, cardiac remodeling, and heart diseases.

## 1. THE HEART IS A SMART PUMP

The heart pumps blood against a mechanical load in every heartbeat. During a cardiac cycle, the cardiomyocytes relax as blood fills the chamber in diastole and then contract to pump the blood into circulation in systole. The right ventricle pumps deoxygenated blood into the lung against a pulmonary vascular resistance, and the left ventricle pumps reoxygenated blood into the whole-body circulation against a much higher systemic vascular resistance. The heart has a remarkable ability to adapt to changing demands of the body. At rest, the systolic blood pressure is about 120 mm Hg but can rise to 300 mm Hg during strenuous exercise (1), and the heart can adjust its force of contraction to overcome this tremendous pressure to eject blood into circulation. Mechanical stress in the heart significantly increases in various heart diseases.

The heart is controlled by extrinsic and intrinsic regulations. Extrinsic autonomic neuroendocrine regulation controls both heart rate and contractile force to maintain cardiac output. Intrinsic autoregulation adjusts the contractile force to control stroke volume without changing heart rate. This review focuses on the intrinsic cellular and molecular mechanisms that sense mechanical strain and stress and transduce to biochemical signals to regulate the contraction of cardiomyocytes in response to load changes.

### 1.1. Introduction

**Figure 1:**
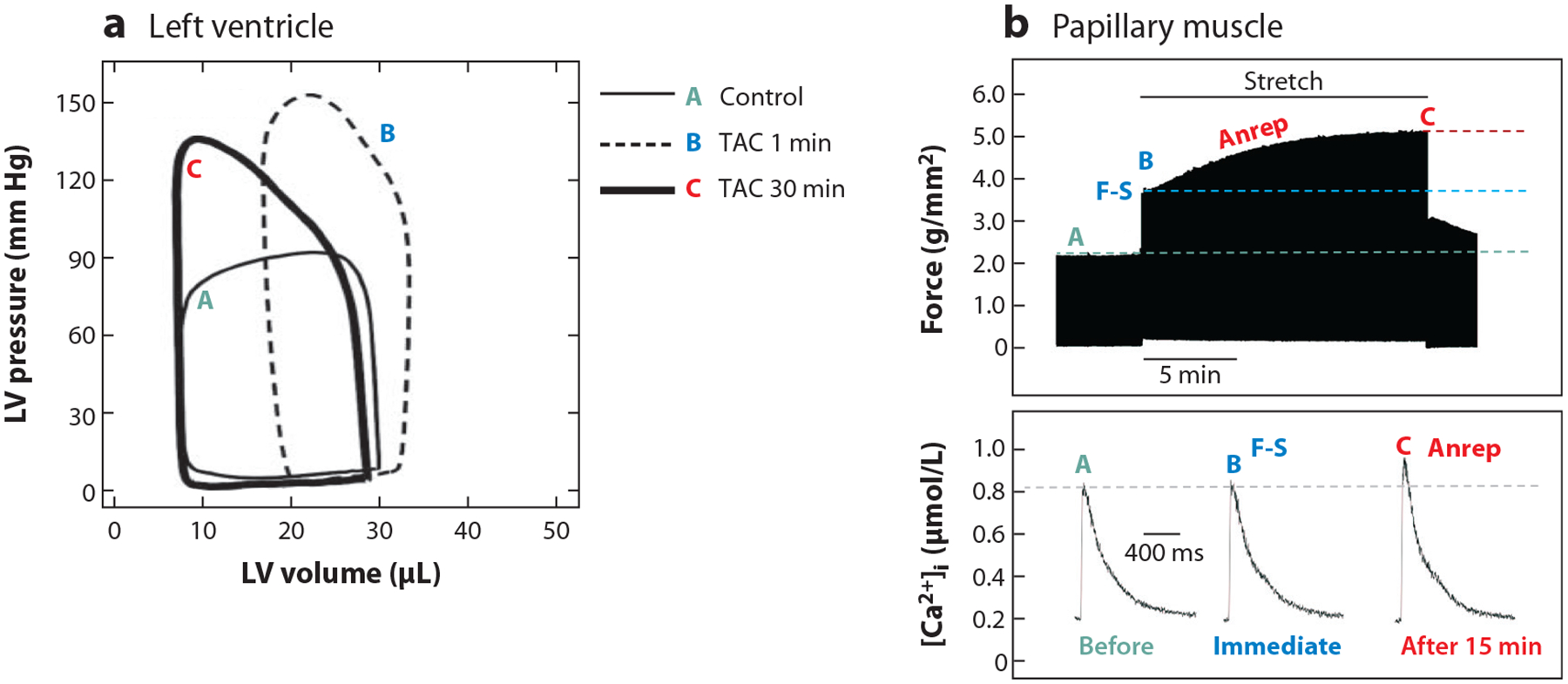
The heart’s intrinsic response to mechanical load: the Frank-Starling (F-S) mechanism and Anrep effect. (*a*) Left ventricular (LV) pressure-volume (PV) loops recorded in mouse heart before and after transverse aortic constriction (TAC). Acute TAC increased the pressure afterload, causing a right shift of PV loop at 1 min after TAC (*dashed line*); notice the enlarged end-diastolic volume (EDV) that engages the F-S mechanism to increase contractility. However, the PV loop shifted left after 30 min (*bold line*). EDV had recovered to near the control value, which would reduce the F-S mechanism; the increased contractility is sustained by the Anrep effect. Panel *a* adapted with permission from Reference 2; copyright 2011 Lippincott Williams & Wilkins. (*b*) Recording of developed force (*top*) and [Ca^2+^]_i_ in trabeculae (*bottom*) before and after stretch. Acute stretch (*horizontal solid line*) causes an immediate increase of force without a change in Ca^2+^ transient (loop A versus loop B). A slow force response developed gradually that is associated with an increase in Ca^2+^ transient (loop C versus loop B). Panel *b* adapted with permission from Reference 46; copyright 1988 American Physiological Society.

The heart must adapt to two situations. First, a change in venous return results in an increase in the left ventricular (LV) end-diastolic volume (EDV), called preload. Second, an increase in the total peripheral resistance increases the afterload. The afterload is the pressure the heart must work against to eject blood from the ventricle into circulation. It is important to remember that an acute increase in afterload reduces the ejection of blood and expands the chamber volume, leading to an increase in preload. This intertwined relationship between afterload and preload is illustrated in the pressure-volume (PV) loop in Figure 1a (2); PV loop A is the control recorded before the acute transverse aortic constriction (TAC); PV loop B is recorded 1 min after TAC that increased the outflow resistance (afterload). Note that reduced blood ejection against the higher resistance resulted in the increase of EDV, which translates to an increase of the cardiomyocyte sarcomere length in the LV wall.^1^ The increase of preload immediately upregulates the contractile force to increase the LV pressure (PV loop B versus PV loop A), which is ascribed to the Frank-Starling (F-S) mechanism.

In Figure 1a, PV loop C is recorded 30 min after TAC. Note that the EDV has returned to near the original value and the LV pressure is as high as in PV loop B. This high pressure is necessary to maintain stroke volume because the outflow resistance is high. Because the EDV has recovered to near the reference value, the force generating the high pressure can no longer be attributed to the F-S mechanism. This slow force response is ascribed to the Anrep effect. Functionally, the F-S mechanism and Anrep effect act in tandem to maintain stroke volume. The F-S mechanism immediately upregulates contractile force upon the initial preload, while the Anrep effect gradually upregulates contractile force in response to sustained afterload.

We start with the discoveries of Otto Frank, Ernest Henry Starling, and Gleb Von Anrep, among others, on the heart’s intrinsic regulation of contractile force in response to load changes (3) and review the subsequent research into the essential features and underlying processes of the F-S mechanism and Anrep effect. We address the interplay and synergy between the F-S mechanism and Anrep effect and highlight the recent research on mechano-chemo-transduction (MCT) mechanisms in the cardiomyocytes. We focus on analyzing the data, consensus and controversies, and open issues in the contemporary literature. We also take a mechano-centric view of heart function and diseases and pose intriguing hypotheses for future investigations. Owing to space limitations, we do not repeat some points already covered by published reviews but simply cite those articles. We also apologize for the limited citations that may miss some research articles that contributed to our collective understanding described herein.

### 1.2. The Frank-Starling Mechanism

The F-S mechanism is named after Otto Frank and Ernest Henry Starling. Although their work set the foundation for understanding the intrinsic regulation of the heart, the essential features were discovered several years prior to Frank’s work at the Carl Ludwig Physiological Institute at the University of Leipzig in Germany (3).

Length-dependent activation is a fundamental property of cardiac muscle. The original observation was made in classic heart-lung preparation (4, 5) and subsequently confirmed in various experimental models at multiple scales including the whole heart, trabecular and papillary muscle (6–9), cardiomyocytes (10–15), and the single myofilament. Presence of length-dependent activation in the single myofilament (16) clearly indicates its origin in the myofilament structure-function. The contribution of the F-S mechanism to cardiac output has been supported by experimental data from both animal models (7, 17–22) and human hearts (23, 24).

In vivo, the F-S mechanism works in conjunction with the autonomic regulation of heart rate and inotropy of cardiac muscle. This inclined some authors to pose the question of whether the F-S mechanism plays any role in the regulation of heart function in situ (25). The modern view holds that the force generation is determined by the synergism of EDV and the contractile state in cardiac muscle (26). Clinical studies revealed another interesting observation that length-dependent activation is reduced or absent in certain pathologies such as heart failure (8, 20, 21, 27–30).

The molecular mechanisms responsible for the F-S mechanism are not well-understood, and it is unlikely that such a fundamental regulatory function has a single mechanism. The first proposed mechanism is the so-called lattice spacing theory. When the cardiomyocyte is stretched along the paralleling running myofilaments in the cell, there should be a reduction in cross-sectional area. Because the number of myofilaments are constant, the spacing between them should be reduced. According to the lattice spacing theory, this proximity results in increased probability of cross-bridge formation and increased Ca^2+^ sensitivity. Therefore, force generation may increase without an increased Ca^2+^ level. This hypothesis was supported by many researchers (31–34), but Konhilas et al. (35) published interesting data contradicting this theory. According to their observation, reduction of interfilament spacing by osmotic compression did not increase Ca^2+^ sensitivity. This notion was challenged in a review article by Moss & Fitzsimons (36), who questioned the reliability of using skinned cardiac muscle as an experimental model by Konhilas et al.

The second proposed mechanism is a change in the overlap of the thick filament and thin filament when sarcomere length is stretched. Sarcomeres at slack length work on the ascending limb of the length-tension curve; therefore, stretching the sarcomere length increases the overlapping zone of the thin filament and thick filament to engage more cross-bridges to generate contractile force. This mechanism may be important at short sarcomere length, but further lengthening of sarcomeres beyond the optimal overlapping zone should reduce the number of cross-bridges toward the descending limb of the length-tension curve. Therefore, this mechanism cannot explain the F-S mechanism at longer sarcomere lengths.

The third proposed mechanism is that Ca^2+^ sensitivity of the contractile machinery increases when sarcomeres are lengthened, which manifests in the leftward shift in the tension-pCa curve. This phenomenon has been reproduced in various experimental models (26, 36, 37). Evidence suggests that cross-bridge formation facilitates the activation of neighboring myosin heads and the cooperation between actin and myosin filaments (37–39). Increased Ca^2+^ sensitivity primarily facilitates cross-bridge formation and force generation but also alters the dynamics of the whole myofilament system. In addition, titin has been proposed to modulate either Ca^2+^ sensitivity or interfilament lattice spacing to contribute to the F-S mechanism (40–45).

### 1.3. The Anrep Effect

The Anrep effect describes the slow force response to an increase in the afterload (46, 47). In response to an acute stretch, there is an almost instantaneous increase of contraction force (see points A and B in the top panel of Figure 1b), which is ascribed to the F-S mechanism and notably independent of any change in Ca^2+^ transient (35, 41). By contrast, the increase in contraction force that underlies the Anrep effect develops slowly over a few minutes (see points B and C in the top panel of Figure 1b) and requires changes in Ca^2+^ transient (48–52) (see points B and C in the lower panel of Figure 1b). Hence, the timescales of the F-S mechanism and Anrep effect differ by an order of magnitude.

We refer the reader to excellent and comprehensive listings of possible molecular mechanisms by Cingolani et al. (46) and Dowrick et al. (47), with the latter giving a useful assessment of each mechanism. Briefly, molecular mechanisms include stretch-activated ion channels (53), stretch-induced angiotensin II (AngII) production (54), AngII-stimulated Na^+^-H^+^ exchanger, reactive oxygen species (ROS) (55, 56), transient receptor potential canonical channel 6 (TRPC6) (57), and thrombospondin 4 (Tsp4) (2). The cellular and molecular mechanisms underlying the Anrep effect are discussed in more detail below.

### 1.4. The Frank-Starling Mechanism and Anrep Effect

Historically, the F-S mechanism and Anrep effect have been viewed as independent processes. We held this view for a long time but now propose that they are not discrete and independent processes but are dynamically linked with a constant interplay between them.

The F-S mechanism activates virtually instantaneously, adapting the developed force to changing vascular resistance or venous return on a beat-to-beat basis. It provides immediate length-dependent activation of the myocardium within 2–3 consecutive cycles. Experiments on muscle fiber also show that the force increases immediately after stretch occurs (see top panel, from points A to B in Figure 1b). As mentioned, this rapid increase in force does not require an increase of Ca^2+^ transient (see lower panel of Figure 1b), which is attributed to the F-S mechanism. Over several minutes, there is a slow increase of force (see top panel, from points B to C in Figure 1b) that is associated with an increase of the Ca^2+^ transient (see bottom panel and compare points C to B in Figure 1b), which is attributed to the Anrep effect. Interestingly, the increase of Ca^2+^ transient amplitude during the slow force response is often subtle compared to the magnitude of the force increase (2, 10, 49–51, 57).

The large change in force could arise from a continually increasing myofilament Ca^2+^ sensitivity, but this is unlikely (50). Instead, we think this large change in force for a small change in the Ca^2+^ transient amplitude reflects the interplay and synergy of the F-S mechanism and Anrep effect. Recall that one posited mechanism underlying the F-S mechanism is the increase of myofilament Ca^2+^ sensitivity (26, 37, 50, 58–60). Under this mechanism, the force would increase even without an increase in Ca^2+^ transient. If the increase in myofilament Ca^2+^ sensitivity involves greater binding of Ca^2+^ to the troponin C in myofilament, then stretching has the equivalent effect of increasing Ca^2+^ buffers. In this case, if the Ca^2+^ transient does not increase, the magnitude of Ca^2+^ transient would decrease (61). But, on balance, the contraction force still increases. As the amount of Ca^2+^ being released from sarcoplasmic reticulum (SR) slowly increases via mechanisms underlying the Anrep effect, the decrease in the cytosolic Ca^2+^ transient would be reversed, and the increase of Ca^2+^ transient amplitude would reach a discernable level. The extra Ca^2+^ would by itself increase the contractile force (62–65). Hence, the F-S mechanism–related increase of myofilament Ca^2+^ sensitivity amplifies the effect of the Anrep-related increase of Ca^2+^ transient.

Not only does the F-S mechanism affect the Anrep effect but the Anrep effect also affects the F-S mechanism. This reverse influence is not seen in the in vitro stretch experiments but is manifest in whole-heart PV loop experiments. PV loop C in Figure 1a arises because the larger Anrep-generated force increases the ejection fraction, so the EDV decreases. The F-S mechanism and Anrep effect interact iteratively in successive cardiac cycles to reduce EDV toward the pre-TAC level.

Note that in Figure 1a, when the EDV had returned to the initial volume after 30 min of TAC, the increase of force can no longer be attributed to the F-S mechanism. The strain (fractional length change) is the same for PV loops A and C but the wall stresses (~pressure × LV radius/LV wall thickness) are different. The mechanisms underlying the increase in contraction force for PV loop C cannot be length-dependent but must be stress-dependent. Thus, the term stress-stimulated contractility coined by Seo et al. (57) is apt.

How can the cardiomyocyte distinguish stress from strain? We proposed a simple model showing how a cardiomyocyte could distinguish stress from strain (66). The model is based on the idea that the cell-surface mechanosensor is coupled to an MCT mechanism involving three elements: cell contraction under three-dimensional (3D) stress, a surface mechanosensor, and intracellular Ca^2+^ transient. We show that this simple, yet general model enables the cardiomyocyte to maintain contraction amplitude despite changes in a range of load. We then confirmed this nontrivial model predictions experimentally (67). Therefore, we hypothesize that the Ca^2+^ regulation is central to how a cardiomyocyte distinguishes stress from strain, but this important question remains an open issue and needs further investigation.

## 2. Ca^2+^ REGULATION IN ADULT VENTRICULAR MYOCYTES UNDER STRAIN AND STRESS

The regulation of systolic Ca^2+^ transient is a key distinction between the F-S mechanism and Anrep effect because the systolic Ca^2+^ transient is unchanged in the former but increased in the latter. Hence, whether and how mechanical strain and stress regulate the Ca^2+^ transient in cardiomyocytes have been a research focus in recent decades. Here, we review the research on how strain and stress regulate the Ca^2+^ signaling in mammalian adult ventricular myocytes (henceforth referred to as cardiomyocytes).

### 2.1. Techniques to Apply Strain and Stress on Cardiomyocytes at the Single-Cell Level

The cardiomyocyte is the basic contractile building block in the heart, as well as the basic unit that integrates a myriad of molecular activities at the whole-cell level to engender the excitation-Ca^2+^ signaling-contraction that governs cardiac function. The cardiomyocyte has a distinct cellular architecture that distributes strain and stress to interconnected subcellular structures throughout the cell. In recent decades, researchers have developed techniques to control strain and stress on cardiomyocytes at the single-cell level. These techniques provide in vitro experimental tools to investigate how different strain and stress affect cardiomyocytes, even though they may not exactly replicate in vivo conditions (which vary immensely). These single-cell techniques can be used in conjunction with modern optical imaging technology to enable detailed studies of cellular and molecular mechanisms of MCT in cardiomyocytes.

#### 2.1.1. 1D stretch.

**Figure 2:**
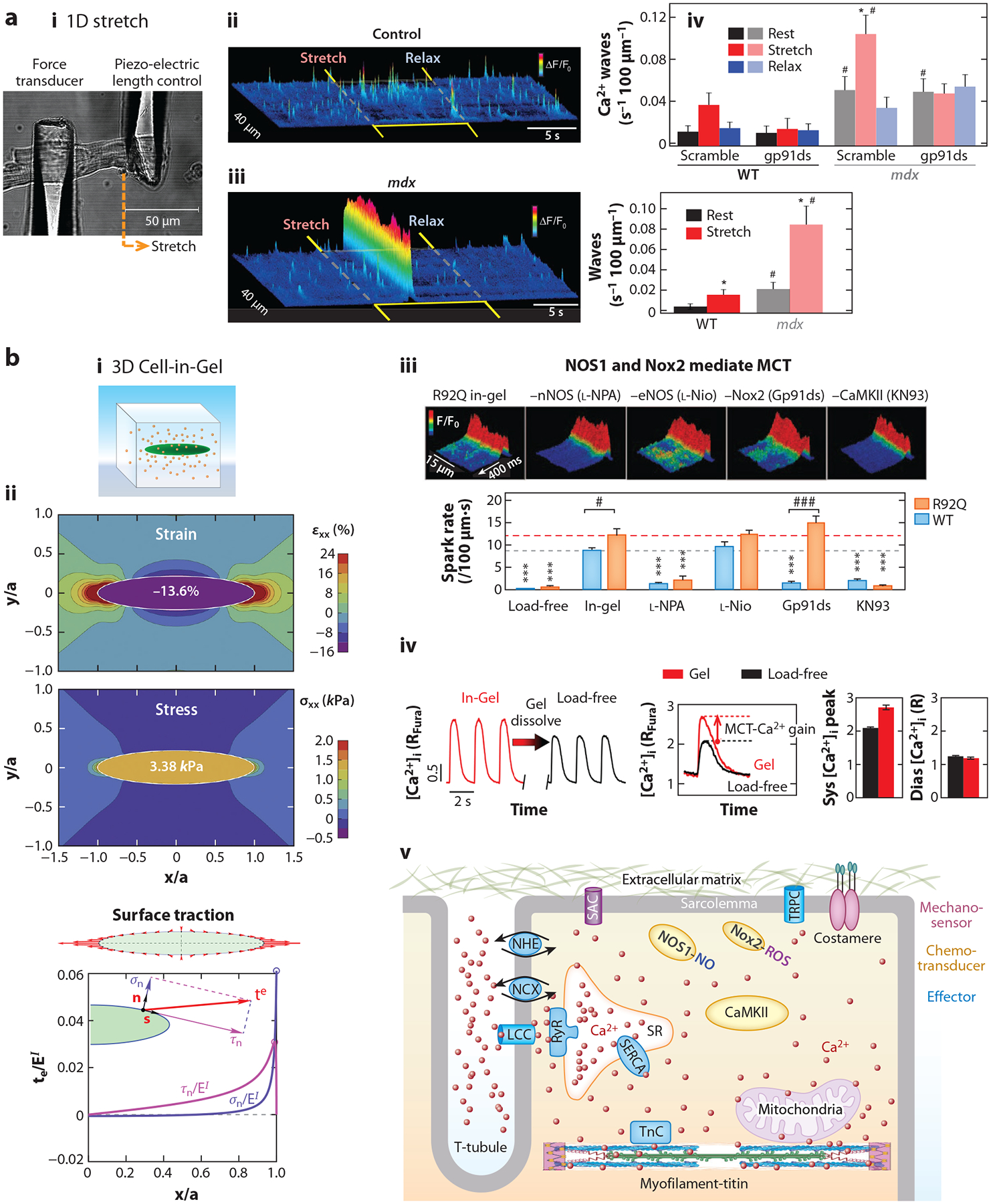
Ca^2+^ regulation by mechanical strain and stress in cardiomyocytes. (*a*) 1D stretch experiments (*a*, *i*) show stretch-/strain-induced Ca^2+^ sparks (*a*, *ii*) and Ca^2+^ wave (*a*, *iii*) in the single cardiomyocytes isolated from WT and *mdx* mouse hearts. (*a*, *iv*) The strain-induced MCT is mediated by Nox2-ROS. Paired *t*-test: *p < 0.05 compared with the rest value, ^#^*p* < 0.05 compared with WT value. (*b*) The 3D Cell-in-Gel technique (*b*, *i*) applies 3D stress on cardiomyocytes (*b*, *ii*). (*b*, *iii*) Stress-induced Ca^2+^ sparks are mediated by NOS1 and Nox2-ROS, but not NOS3. (*b*, *iv*) Stress-induced increase of the Ca^2+^ transient, called MCT-Ca^2+^ gain. (*b*, *v*) Molecules involved in the stress-induced MCT that regulate the Ca^2+^ signaling system. The two-way ANOVA shows significant difference in the spontaneous Ca^2+^ spark rate between the R92Q and WT (*p* < 0.0001), significant drug effects (*p* < 0.0001), and significant interaction (*p* < 0.0001). The Bonferroni posttest shows significant difference for the drug effect compared to Cell-in-Gel without drug (***p < 0.001) on each genotype, a significantly higher spark rate in R92Q cardiomyocytes than in WT cardiomyocytes for the Cell-in-Gel condition (^#^*p* < 0.05), and the Gp91ds effect (^###^*p* < 0.001). Abbreviations: 1D, one-dimensional; 3D, three-dimensional; CaMKII, Ca^2+^/calmodulin protein kinase II; Ctrl, control; Dias, diastolic; LCC, L-type Ca^2+^ channel; MCT, mechano-chemo-transduction; l-Nio, *N*^5^-(1-iminoethyl)-l-ornithine dihydrochloride; l-NPA, *N*^ω^-propyl-l-arginine hydrochloride; NCX, Na^+^/Ca^2+^ exchanger; NHE, Na^+^/H^+^ exchanger; NOS, nitric oxide synthase; Nox, nicotinamide adenine dinucleotide phosphate oxidase; R, fluorescence ratio; RyR, ryanodine receptor; SAC, stretch-activated channel; SERCA, sarcoendoplasmic reticulum Ca-ATPase; Sys, systolic; TnC, troponin C; TRPC, transient receptor potential canonical channel; WT, wild-type. Panel *a* adapted with permission from Reference 69; copyright 2011 AAAS. Panel *b*, *i* and *iii* adapted with permission from Reference 70 (CC BY-NC-ND 4.0); panel *b*, *ii* (top two subpanels) adapted with permission from Reference 72 (CC BY-NC-ND 4.0) and panel *b*, *ii* (bottom subpanel) adapted with permission from Reference 71 (CC BY-NC-ND 4.0); panel *b*, *iv* (left two subpanels) based on data from Reference 67 and panel *b*, *iv* (right two subpanels) adapted with permission from Reference 67, copyright 2021 Lippincott Williams & Wilkins; panel *b*, *v* is an original drawing by A. Domala, S. Pagay, and Y. Chen-Izu. Part of the image created using BioRender.com.

In the late 1980s, researchers developed one-dimensional (1D) stretch techniques by attaching a pair of microcantilevers to the surface of a rod-shaped single cardiomyocyte to stretch the cell segment. Le Guennec et al. (68) first introduced the 1D stretch technique using carbon fibers to apply preload by stretching. The compliant carbon fibers were also used to apply afterload by resistive bending during auxotonic cell contraction (13). A variation of the technique replaced carbon fibers with rigid glass rods to stretch the cell (69). The 1D stretch technique is used to apply longitudinal tension in the cardiomyocyte but does not apply lateral compression or surface traction because the cell is bathed in aqueous solution (Figure 2a, **subpanel *i***).

#### 2.1.2. 3D Cell-in-Gel.

In the 2010s, researchers invented new techniques to apply 3D mechanical load on single cardiomyocytes to better mimic the in situ environment. Chen-Izu’s group developed the Cell-in-Gel technology to embed cardiomyocytes in a 3D viscoelastic hydrogel (Figure 2b, **subpanel *i***) (70). The stress levels can be tuned by the hydrogel stiffness. Mechanical analyses show that the cardiomyocyte contracting in-gel experiences 3D stress: longitudinal tension, transverse compression, and surface tractions with normal and shear stress (71–73) (Figure 2b, **subpanel *ii***; Supplemental Videos 1–3). Importantly, because cardiomyocytes are embedded in gel at the slack length without pre-stretch, the Cell-in-Gel technique is well-suited for investigating the afterload effects on cardiomyocytes without the confounding influence of preload.

#### 2.1.3. Other techniques.

Controlling mechanical load at the single-cell level is often technically challenging. Besides the 1D stretch and 3D Cell-in-Gel techniques, researchers have also made considerable efforts to develop other techniques. For example, Petroff et al. (74) embedded single cardiomyocytes in agarose and then encased the cell-in-agar within a silicon tube. Because agarose does not adhere to the cell surface, stretching the tube should squeeze the cell-in-agar to apply transverse compression rather than longitudinal strain. A variation was to use fibrin instead of agarose (15). The two-dimensional (2D) stretch techniques use deformable membrane to attach to (cultured) cells, and stretching the membrane applies tension and shear stress on the cells (75). Nevertheless, the 2D method requires cell adhesion to the membrane, which can be used for cultured (embryonic/neonatal) immature cardiomyocytes, but it is impractical for rod-shaped cardiomyocytes.

#### 2.1.4. Different experimental techniques apply different mechanical strain and stress in the cardiomyocyte architecture.

It is important to know the distribution of strain and stress in the various stress-bearing structures of the cell when comparing the results obtained from different experimental techniques. Otherwise, confusion may arise on the apparent discrepancies from different experiments. For example, cell-surface traction is present in the 3D Cell-in-Gel setting but absent in the 1D stretch setting. It is conceivable that surface mechanosensors are engaged in the 3D Cell-in-Gel experiments but not the 1D stretch experiments, leading to different MCT pathways. On the other hand, different techniques can be utilized strategically to dissect the different strain and stress in distinct subcellular structures and to investigate their respective MCT pathways.

### 2.2. Ca^2+^ Regulation in the Cardiomyocytes Under 1D Mechanical Strain

In the 1970s and 1980s, researchers developed methods to use multicell preparations such as ventricular trabeculae and papillary muscle to simultaneously measure the Ca^2+^ transient and developed force under controlled strain and stress. A classic experiment is to rapidly stretch the muscle bundle to a set sarcomere length and measure the developed isometric tension. Such experiments show that stretching the muscle bundle causes an immediate increase of the contractile force within seconds, which is followed by a slow increase of the force over minutes (called slow force response) (2, 49, 50, 52, 76). The consensus is that strain-induced rapid increase of force is myofilament based and does not involve changes in the Ca^2+^ transient (2, 36, 52, 77). By contrast, the slow force response involves increases of Ca^2+^ transient and, in some cases, increased myofilament Ca^2+^ sensitivity; both contribute to enhance force generation (2, 49, 50, 52).

Since the 1990s, researchers have used the single-cell techniques in conjunction with modern optical imaging technology to perform in-depth studies of the cellular and molecular mechanisms of mechanosensing and mechanotransduction. Using the 1D stretch with carbon fibers, Iribe et al. (13) performed rigorous experiments to study the force-length relationship in auxotonic, isotonic, isometric, and work loop contractions in ventricular myocytes. They found that “a cellular equivalent of the Frank-Starling effect could be observed in all cases: an increase in preload (diastolic length) leads to an increased ability to perform work” (13, p. H1493). However, they also found that “effects of slow force responses to stretch are negligible in this setting” (13, p. H1492). Note that this new finding by 1D stretch of an isolated single cardiomyocyte is different from the presence of slow force response by stretching multicell preparations. Perhaps this apparent discrepancy provides an important clue for deciphering the involvement of different mechanosensors in different experimental settings. Seo et al. (52) performed comprehensive experiments studying afterload effects in the intact heart and trabeculae and at single-cell levels. Using 1D stretch with carbon fibers, they found that stretching the cardiomyocyte during auxotonic contraction caused an immediate increase of developed force without any change in the Ca^2+^ transient, followed by a slow increase of force associated with a (slight) increase of the Ca^2+^ transient (called stress-stimulated contractility). Their findings at the single-cardiomyocyte level point to the cellular origin of the F-S mechanism and Anrep effect.

1D stretch of cardiomyocytes also gives rise to an increase of spontaneous Ca^2+^ sparks (69, 78) (Figure 2a, **subpanel *ii***). This might be caused by changes in the SR Ca^2+^ content because axial stretch enhanced the rate of rest decay and reloading of SR Ca^2+^ (79). Moreover, the stretch-induced Ca^2+^ sparks are augmented in *mdx* mouse cardiomyocytes, a muscular dystrophy model with dystrophin replaced by utrophin (69) (Figure 2a, **subpanel *iii***). This suggests a tentative role of dystrophin in the mechanotransduction pathway. Furthermore, Prosser et al. (80) discovered that stretch-induced Ca^2+^ sparks were abolished by inhibiting the nicotinamide adenine dinucleotide phosphate oxidase 2 (Nox2) but not Nox4 (Figure 2a, **subpanel *iv***). Therefore, Nox2-generated ROS signaling is a critical mediator of the stretch effect. They also showed that, while a single stretch elicited only a transient burst of ROS, repetitive stretch-shortening cycles elicited a sustained increase of ROS production and high Ca^2+^ spark frequency (80). Taken together, these studies identify Nox2 as an essential chemotransducer in the MCT pathway activated by stretch. It is plausible that internal mechanosensor(s) may sense axial stretch and activate Nox2, but the molecular identity of the mechanosensor remains unclear.

### 2.3. Ca^2+^ Regulation in the Single Cardiomyocyte Under 3D Mechanical Stress

The Cell-in-Gel experiments show a significant increase of the systolic Ca^2+^ transient and diastolic Ca^2+^ sparks in the cardiomyocytes contracting under 3D stress (67, 70, 81) (Figure 2b, **subpanels *iii, iv***). To decipher the molecular mechanism, we further developed the Patch-Clampin-Gel technique to do electrophysiology recordings in the cardiomyocyte in-gel (82). We found that cardiomyocytes contracting under afterload show increased L-type Ca^2+^ current density, decreased inward rectifier K^+^ current density, and prolonged action potential duration, among other changes (82). The total amount of Ca^2+^ entry into the cell during the cardiac cycle should be increased by a larger L-type Ca^2+^ current and longer action potential duration, which may elevate the SR Ca^2+^ content and increase the SR Ca^2+^ release, leading to increased cytosolic Ca^2+^ transient. The increase of peak Ca^2+^ transient in the cardiomyocyte under mechanical load (versus load-free) by MCT is quantified as the MCT-Ca^2+^ gain (67, 70) (Figure 2b, **subpanel *iv***).

Furthermore, all the abovementioned stress-induced effects are abolished by inhibiting the nitric oxide (NO) synthase (NOS), specifically by inhibiting NOS1 (nNOS) but not NOS3 (eNOS) (70, 82). The stress-induced Ca^2+^ sparks were also abolished by inhibiting Nox2 (70). ROS and NO signaling can modulate many molecules including Ca^2+^/calmodulin protein kinase II (CaMKII) (83, 84). Indeed, pharmacological inhibition of CaMKII or genetic deletion of the *S*-nitrosylation site on CaMKII (CaMKII-C290A) effectively abolished stress-induced increases of Ca^2+^ sparks and Ca^2+^ transient (70, 85). Therefore, NOS1, Nox2, and CaMKII are essential chemotransducers in the 3D stress–activated MCT pathway. These important enzymes should modulate many downstream effector molecules involved in regulating the cardiomyocyte excitation-Ca^2+^ signaling-contraction (Figure 2b, **subpanel *v***). The upstream mechanosensor(s) that sense the stress and activate the chemotransducers are yet to be identified.

## 3. MECHANO-CHEMO-TRANSDUCTION PATHWAYS

Development of innovative techniques to control mechanical load at the single-cell level enabled in-depth studies of how strain and stress affect the regulation and function of cardiomyocytes. Research has been gaining momentum to decipher the MCT pathways that transduce mechanical load to biochemical signals to regulate excitation-Ca^2+^ signaling-contraction. These pathways have been the subject of excellent reviews (46, 86–88). We will not repeat those reviews but propose a new conceptual framework to reorganize the existing data. The goal is to decipher how various strain and stress on cardiomyocytes may engage different mechanosensors and their respective MCT pathways to modulate the cell’s responses to various mechanical stimuli.

The heart has a complex mechanical structure and is subjected to dynamic loading in every heartbeat. At the organ level, preload and afterload exert 3D strain and stress in myocardium. At the cell level, cardiomyocytes experience various strain and stress depending on the anatomic location and the contractile state. At the molecular level, mechanosensors in various subcellular structures experience different strain and stress. To delineate the MCT pathways, we need to (*a*) know the 3D distribution of the strain and stress field in the cardiomyocyte architecture, (*b*) identify the mechanosensors in various stress-bearing structures, and (*c*) decipher MCT pathways from mechanosensors to chemotransducers and to effector molecules.

Like constructing a jigsaw puzzle from pieces, it helps to sort the molecules involved in mechanotransduction into different provisional groups: (*a*) structural elements (e.g., collagen, microtubule, desmin, lamin) that are load-bearing and affect the cellular mechanics, which would broadly alter many cellular processes to impair function; (*b*) mechano-sensitive molecules (e.g., stretch-activated channels, piezo channels) that are directly modulated by strain and stress; and (*c*) mechanosensors (e.g., integrin, dystroglycan) that link to chemotransducers to regulate downstream effectors. According to their locations in the cell architecture, mechanosensors can be subgrouped into internal or surface mechanosensors, which detect the local strain and stress in their respective subcellular environments and engage different MCT pathways.

### 3.1. The Cardiomyocyte Architecture and Structural Elements

**Figure 3:**
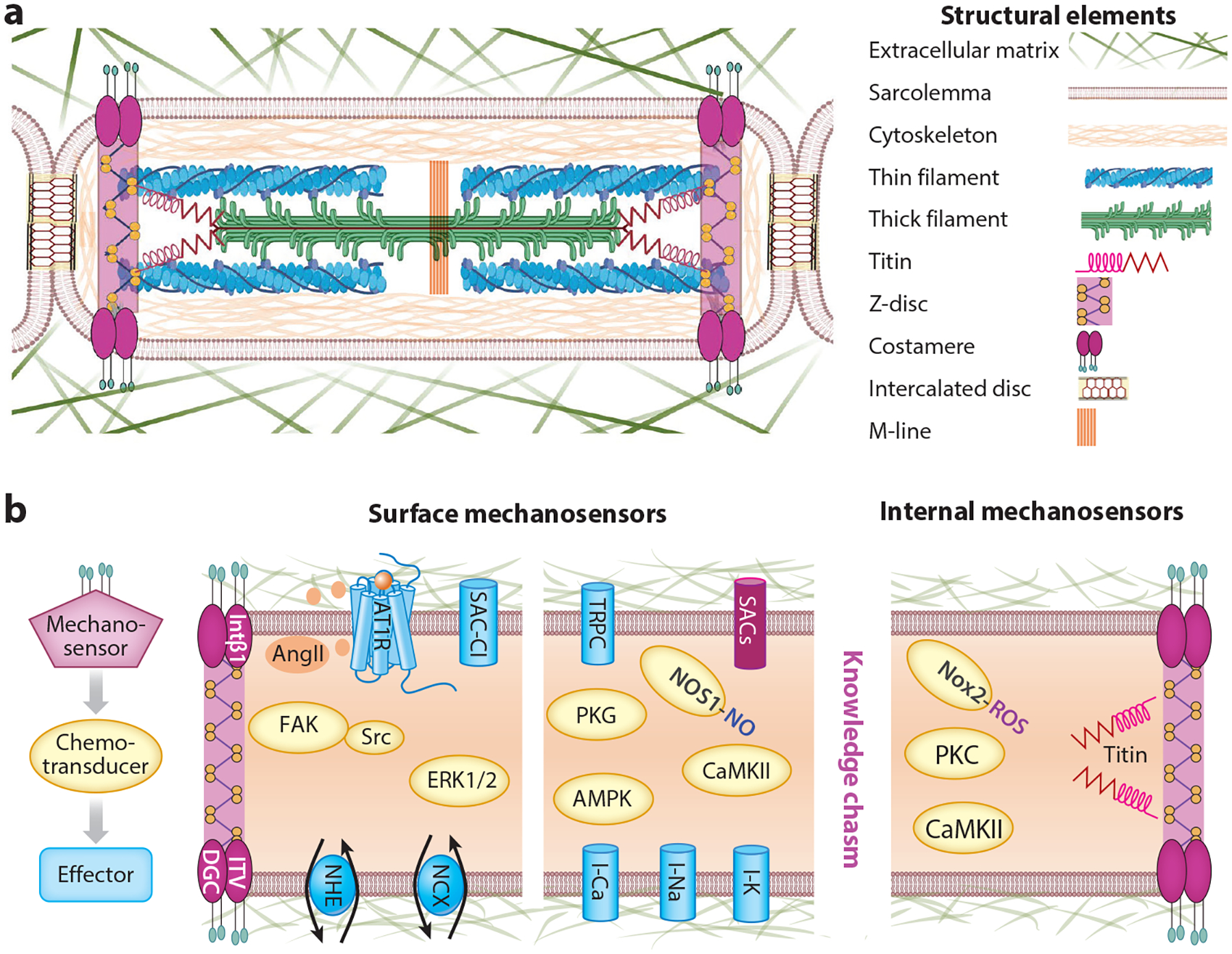
Schematic of cardiomyocyte structure and MCT pathways. (*a*) Conceptual schematic of the cardiomyocyte architecture and the structural elements; one sarcomere is depicted as the basic unit in the repeating structure of the cell. (*b*) Conceptual schematic of MCT pathways comprising mechanosensors (*magenta*), chemotransducers (*yellow*), and effectors (*blue*). The surface mechanosensors sense the strain/stress at the cell–extracellular matrix interface; internal mechanosensors sense the strain/stress within the cell. Shown are some molecules involved in MCT, but knowledge gaps exist in the field. Abbreviations: AngII, angiotensin II; AT1R, angiotensin II type I receptor; CaMKII, Ca^2+^/calmodulin kinase II; DGC, dystrophin glycoprotein complex; ERK1/2, extracellular signal–regulated kinases 1 and 2; FAK, focal adhesion kinase; INTβ1, integrin-β1; ITV, integrin-talin-vinculin complex; LCC, L-type Ca^2+^ channel; MAPK, mitogen-activated protein kinase; MCT, mechano-chemo-transduction; NCX, Na^+^/Ca^2+^ exchanger; NHE, Na^+^/H^+^ exchanger; NO, nitric oxide; NOS, nitric oxide synthase; Nox, nicotinamide adenine dinucleotide phosphate oxidase; PKA, protein kinase A; PKC, protein kinase C; PKG, protein kinase G; ROS, reactive oxygen species; RyR, ryanodine receptor; SAC, stretch-activated channel; SAC-Cl, stretch-activated chloride channel; SERCA, sarcoendoplasmic reticulum Ca-ATPase; TnC, troponin C; TnI, troponin I; TRPC, transient receptor potential canonical channel. Original drawings by A. Domala, S. Pagay, and Y. Chen-Izu. Parts of the image created using BioRender.com.

The cardiomyocyte has a unique cellular architecture with distinct mechanical load-bearing and force-transmitting ultrastructure. As illustrated in Figure 3a, the active force generation for muscle contraction is produced by the myofilament comprising the thick filament and thin filament. The myofilament is anchored by Z-discs flanking the sarcomere. The titin-spanning half of the sarcomere links across the Z-disc, I-band, A-band, and M-line. The cytoskeleton network cross-links various structural elements including intermediate filaments, microtubules, and actin filaments that form a tensegrity-like structure that distributes mechanical stress throughout the cell (89, 90). The cell-surface plasma membrane also transmits force to embedded proteins (91, 92). The extracellular matrix (ECM) binds to many cell-surface molecules and mechanically connects the neighboring cells. In vivo, cardiomyocytes are connected end-to-end through the intercalated disc (cell–cell adhesion) (93) and side-by-side through the ECM (cell–ECM adhesion) (94) to transmit mechanical stresses across the myocardium.

Along the longitudinal axis of a cardiomyocyte, passive tension is generated mainly by the titin filament, intermediate filament, and microtubules (95). The cell’s viscoelasticity is also tuned by the various isoform expression and posttranslational modification of structural molecules (96). Along the lateral axis of a cardiomyocyte, a major stress-bearing structure is the costamere, comprising the dystrophin glycoprotein complex and integrin-talin-vinculin complex. The costamere protein complex connects the Z-disc, cytoskeleton, sarcolemma, and ECM (97). The costamere and Z-disc form truss-like structures that transmit force across the intracellular structures and the ECM (98). Bloch & Gonzalez-Serratos (99) found that approximately 70–80% of force transmission is supported by the lateral mechanical scaffolding in the skeletal myocytes (a similar structure is found in cardiomyocytes).

Alteration of structural elements may change the mechanical properties and disrupt force transmission in myocardium, leading to cardiac dysfunction and heart diseases. For example, microtubules in the cytoskeleton undergo posttranslational modification in heart failure, which contributes to increased cardiomyocyte stiffness, impaired cellular trafficking, and contractile dysfunction (96). Desmin and lamins in the cytoskeleton transmit mechanical force to the nucleus and sarcolemma, and their mutations and posttranslational modifications affect the transcription of many molecules, leading to severe cardiomyopathies (100). Titin isoform switch and posttranslational modifications occur in various forms of heart diseases, contributing to increased myocyte stiffness and impaired contraction in heart failure (101, 102). In dilated cardiomyopathy, remodeling of ECM and fibrosis increases the myocardium stiffness but also provides structural reinforcement for a weakened heart (103, 104). Extensive studies of the structural elements and their involvement in cardiac dysfunction and heart diseases have been described in excellent reviews (53, 96, 100–104). We do not repeat those reviews but focus on the MCT pathways that link mechanosensors to chemotransducers to regulate the Ca^2+^ transient.

### 3.2. Mechanosensors and Mechano-Chemo-Transduction Pathways

Understanding mechanotransduction mechanisms requires mapping out the MCT pathways from mechanosensors to chemotransducers and to effector molecules. Here, we review some putative MCT pathways in the literature, recognizing that this field is still in a pioneering stage with many uncharted pathways.

#### 3.2.1. Titin.

Spanning half of the sarcomere in parallel to the myofilament, titin is a giant multitasking protein that serves as a principal structural element, a regulator of passive and active tension, and a putative internal mechanosensor (102, 105). Titin filament links the Z-disc to the I-band, A-band, and M-line. Titin’s I-band segment is variable, extensible (105), and well-suited to sense axial stretch. Different isoforms of titin have different compliance and spring-like properties (105). Titin’s stiffness is also regulated by posttranslational modifications including acetylation, oxidation, and phosphorylation (106). Although titin is regulated by kinases and phosphatases including protein kinase A (PKA) (107) and CaMKII (108, 109), little is known about whether titin transmits force to regulate the enzyme activities. Thus far, whether titin transduces force to modulate a specific MCT pathway is unresolved and warrants further investigation.

#### 3.2.2. Extracellular matrix–Tsp4.

The ECM binds to many cell-surface molecules and mechanically connects the neighboring cells in the tissue to transmit force (Figure 3b). Studies show that ECM plays an essential role in activating the Anrep effect. Disrupting ECM by deleting Tsp4, which is a cross-linker of proteins in ECM (110), effectively abolishes the Anrep effect (2). In the heart under hypertension, pressure overload, or myocardial infarction (111, 112), Tsp4 is rapidly upregulated, indicating its important role in stress-related adaptive response. Kass’s group (2) created the Tsp4^−/−^ knockout mouse and studied the PV loop changes following TAC. They found that the Tsp4^−/−^ heart had a typical F-S mechanism response but no Anrep effect. This indicates the essential involvement of the ECM in the MCT underlying the Anrep effect.

##### 3.2.2.1. Integrin-AngII pathway.

As a part of the costamere, integrins on the cell surface are glycosylated and attached to the collagen/fibronectins in ECM (113, 114) (Figure 3b). Browe & Baumgarten (115) show that applying force on the cell-surface integrin-β1 protein leads to secretion of AngII from cardiomyocytes. Then the autocrine AngII signaling activates the AngII type 1 receptor (AT1R), which is a G protein–coupled receptor that leads to a signaling cascade to modulate the stretch-activated chloride channel. Other studies also show that AngII signaling activates the Na^+^/H^+^ exchanger and the reverse mode Na^+^/Ca^2+^ exchanger (46, 51, 116), which may increase the intracellular Ca^2+^ concentration. Moreover, AT1R activation is found to mediate the slow force response in cat papillary muscle (117). These data suggest an MCT pathway that connects ECM to the integrin-β1 and to the autocrine AngII signaling, which in turn modulates ion channels and exchangers, leading to regulation of the intracellular Ca^2+^ transient in cardiomyocytes.

##### 3.2.2.2. Dystrophin-TRPC6 pathway.

As another part of the costamere, the dystroglycans on the cell surface are also attached to ECM and transmit mechanical force inwardly to dystrophin (118) (Figure 3b). Genetic defects in dystrophin cause Duchenne muscular dystrophy (DMD) that manifests as severe neuromuscular defects and cardiac arrhythmias (119). DMD hearts are more susceptible to pressure overload than are healthy hearts (120). TRPC6 is upregulated in DMD (52) and in other pressure overload heart failure (121, 122). Kass’s lab (52) performed comprehensive studies using dystrophin and utrophin knockout (*mdx*/*utrn*^+/−^, *mdx*/*utrn*^−/−^) and Trpc6 knockout (Trpc^−/−^) mouse models. They found that the stress-induced Ca^2+^ transient increase and slow force response are hyperactivated in the *mdx*/*utrn*^+/−^ and *mdx*/*utrn*^−/−^ models but suppressed in Trpc^−/−^ models. Furthermore, the exacerbated stress response in the DMD models can be suppressed by inhibiting TRPC6 activity by pharmacological inhibitors or by guanosine 3′,5′-cyclic monophosphate–activated protein kinase G (cGMP-PKG) phosphorylation of TRPC6 (52). These data suggest an MCT pathway that links the dystrophin glycoprotein complex to TRPC6, leading to the increased Ca^2+^ transient and slow force response to afterload. However, it remains unknown whether TRPC6 is activated directly by the dystrophin glycoprotein complex or through a chemotransducer.

##### 3.2.2.3. Mechanosensitive ion channels.

Mechanosensitive ion channels in the cell membrane are logical candidates for sensing mechanical stress (Figure 3b). Many ion channels are glycosylated in the extracellular domains and most likely attached to ECM (123) and thus exposed to external force. Ion channels are also attached to the cytoskeleton (124) and thus subjected to internal force. Mechanosensitive ion channels can be classified into two groups: mechano-gated channels and mechano-modulated channels (125). The mechano-gated channels are activated by mechanical stimuli alone. This group includes stretch-activated channels (SACs) and volume-activated channels. Mechano-modulated channels are gated by other stimuli such as voltage or a ligand but are modulated by mechanical stress. This group includes voltage-gated Ca^2+^ channels, Na^+^ channels, and K^+^ channels. Given that ion channel gating and permeability are controlled by the protein structure, it is not surprising that external force on the channel protein may cause structural deformation that affects the channel gating kinetics and open probability.

The stretch-activated nonselective cation channel (SAC_ns_) is Ca^2+^ permeable and may contribute to increased Ca^2+^ influx into cardiomyocytes. SAC_ns_ activation is also expected to depolarize the cell membrane (126). The stretch-activated K^+^ channel (SAC_k_) activation would cause K^+^ efflux and is expected to repolarize the cell membrane (127). Piezo1 and Piezo2 are SACs found in the murine heart with low-level messenger RNA (mRNA) (128). The protein expression and functional measures of Piezo1 and Piezo2 to demonstrate their role in cardiomyocytes have not been established (124, 125). Thus far, we found one study showing that cardiac-specific knockout or overexpression of Piezo1 altered the stretch-induced ROS signaling, Ca^2+^ sparks, and caffeine-sensitive Ca^2+^ release in cardiomyocytes. Nonetheless, the Piezo1 current was not directly measured but inferred from the effect of its activator Yoda (129). The cardiac-specific Piezo2 knockout mice did not show any morphological abnormalities or altered cardiac function under normal conditions (130).

##### 3.2.2.4. Nox2-ROS pathway.

The ROS produced by NADPH oxidase (Nox) is found to mediate the stretch-induced Ca^2+^ sparks in cardiomyocytes (Figure 3b). Kohl’s group (78) and Lederer’s group (69) discovered that 1D stretch causes a transient increase of spontaneous Ca^2+^ sparks in resting cardiomyocytes. Prosser et al. (69) further showed that Nox2- (but not Nox4)-derived ROS are necessary for the stretch response, indicating Nox2 as an essential chemotransducer in the strain-activated MCT pathway. Furthermore, disruption of microtubules abolished stretch-induced Ca^2+^ sparks (69, 78, 131, 132), indicating a role of the cytoskeleton in transmitting mechanical force to activate the Nox2-ROS signaling. A candidate for the mechanosensor might be integrin-β1 that leads to autocrine AngII signaling to activate the sarcolemmal Nox (115). Nonetheless, in the 1D stretch experiment, the cell surface is exposed to bath solution, and it is unclear how the integrin-β1 would be under stress and how the secreted AngII would not be washed away by continuous perfusion of the bath solution. Hence, the molecular identity of the mechanosensor upstream from the stretch-activated Nox2-ROS pathway remains unknown and warrants further investigation.

##### 3.2.2.5. NOS1-NO pathway.

The NO signaling plays a significant role in stress response (Figure 3b). Produced by NOS, NOS-NO signaling was found to be activated by compressing the cardiomyocyte encased in agarose (74). Using the Cell-in-Gel system to apply 3D stresses on the cardiomyocytes during auxotonic contraction, Jian et al. (70) found that NOS-NO signaling critically mediates stress-induced increases of systolic Ca^2+^ transient and diastolic Ca^2+^ sparks. Specifically, pharmacological inhibition or genetic deletion of NOS1, but not NOS3, abolished the stress-induced changes in Ca^2+^ transients and Ca^2+^ sparks. Subsequent studies showed that inhibiting NOS1 also abolished stress-induced effects on ionic currents and the action potential (82). More evidence comes from the 2D stretch experiment by Garbincius & Michele (133) showing that repetitive stretching of the cardiomyocytes attached to a deformable 2D membrane increased the intracellular NO concentration. They found that AMP-activated protein kinase (AMPK) mediates the NOS1 phosphorylation. These data consistently show that NOS1-NO signaling is an essential chemotransducer in the stress-activated MCT pathway. However, the mechanosensor upstream from the stress-induced NOS1-NO pathway is not yet known and warrants further investigation.

### 3.3. Apparent Discrepancy and an Intriguing Hypothesis

Thus far, studies from several labs show some seemingly discordant results. In the 1D stretch experiments, using l-NAME to inhibit NOS indicated that NO signaling is not involved in the 1D stretch–induced Ca^2+^ response in mouse or rat cardiomyocytes (51, 69, 78). By contrast, the Cell-in-Gel experiments showed that NOS1-NO signaling is essential for mediating the 3D stress–induced response in mouse and rabbit cardiomyocytes (67, 70, 82). The intracellular NO level is also found to be increased by repetitively stretching the cardiomyocytes attached to the 2D elastic membrane (133). We think the discrepancy could be because different experimental settings apply different strain and stress on subcellular structures, which may activate different mechanosensors at different locations. Quantitative mechanical analyses show that the cell surface is exposed to bath solution with little stress in the 1D stretch setting, but it is adhered to ECM and subjected to surface traction (normal and shear stresses) in the 2D stretch or 3D Cell-in-Gel settings. It is plausible that the cell-surface mechanosensors are engaged in the 2D and 3D experiments to activate the NOS1-NO pathway but are uninvolved in the 1D stretch experiments. In contrast, the internal mechanosensors are engaged during cell contraction in all these experimental settings. Indeed, Nox2-ROS signaling is found to be activated in both the 1D stretch (69) and 3D Cell-in-Gel (70) experiments. Piecing together the data from the 1D, 2D, and 3D experiments, we think a pattern emerges to suggest an intriguing hypothesis that the surface mechanosensors activate the NOS1-NO pathway and possibly the Nox2-ROS pathway, whereas the internal mechanosensors activate the Nox2-ROS pathway but not the NOS1-NO pathway (87).

### 3.4. Plurality of Mechanosensors and Network of Mechano-Chemo-Transduction Pathways

Given the diverse mechanical stimuli and the complex cardiomyocyte ultrastructure, we expect a plurality of mechanosensors located in distinct locations in the cell architecture. The mechanosensors are linked upstream to stress-bearing structures and downstream to their respective MCT pathways. Detailed knowledge on the 3D strain and stress distribution in the cell architecture will inform the location and identity of mechanosensors, their links to specific chemotransducers, and the downstream effectors that regulate the cell function. Thus far, only a few MCT pathways have been studied. Identifying various mechanosensors and mapping their MCT pathways presents a new research frontier, which is fundamentally important for understanding the heart’s intrinsic regulation.

MCT pathways lead to cascading NO signaling and ROS signaling that are known to regulate the activities of many enzymes such as CaMKII and PKA. Moreover, the NO and ROS signaling pathways also interact with other major signaling pathways. For example, in heart failure, cardiomyocytes are exposed to abnormal β-adrenergic stimulation (134) and increased mechanical stress (135). β-Adrenergic signaling also links to PKA, CaMKII (136), NO (137), and ROS (138, 139). To gain a comprehensive view, it will be important to learn how these pathways cross talk and how they constitute a signaling network to orchestrate coordinated cell responses to varying load conditions for the purpose of maintaining cardiac output.

## 4. AUTOREGULATION OF EXCITATION-Ca^2+^ SIGNALING-CONTRACTION COUPLING AND ARRHYTHMOGENIC ACTIVITIES IN CARDIOMYOCYTES UNDER MECHANICAL LOAD

### 4.1. Autoregulation of Excitation-Ca^2+^ Signaling-Contraction in Response to Load Changes

**Figure 4:**
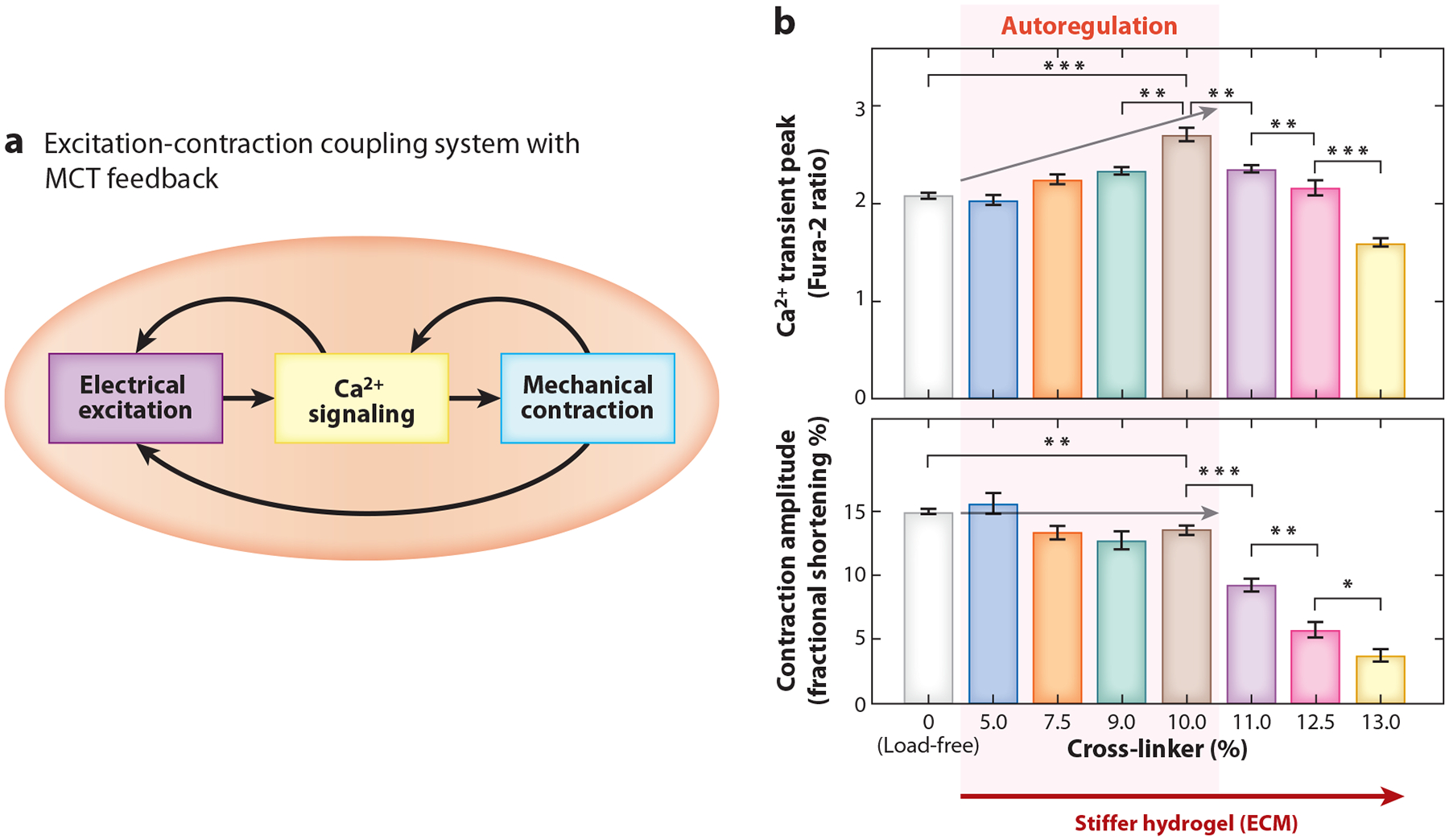
Autoregulation of excitation-Ca^2+^ signaling-contraction by closed-loop MCT feedback. (*a*) Schematic of the excitation-contraction coupling system with MCT feedback, which enables autoregulation. (*b*) The MCT mechanism regulates the Ca^2+^ transient in a load-dependent manner. The cell contraction amplitude is maintained by increases of MCT-Ca^2+^ gain despite the increasing load in a range but fails in overload condition. Gray arrows highlight the direction of change with increasing cross-linker concentration. Abbreviations: ECM, extracellular matrix; MCT, mechano-chemo-transduction. Student’s *t*-test: *p < 0.05, **p < 0.01, ***p < 0.001. Figure adapted with permission from Reference 67; copyright 2021 Lippincott Williams & Wilkins.

In a working heart, the cardiomyocytes perform beat-to-beat excitation, Ca^2+^ signaling, and contraction under mechanical load. The classical paradigm of cardiac excitation-contraction (E-C) coupling involves a forward flow of information in the three dynamical systems: The electrical system generates an action potential, triggering Ca^2+^ signaling to produce a Ca^2+^ transient, which then activates the myofilament to cause a contraction (Figure 4a). Recent studies show that cardiomyocytes also have MCT feedback pathways (Figure 4a). Furthermore, cardiomyocytes are capable of autoregulating MCT-Ca^2+^ gain in a certain range of load changes, but this mechanism fails under mechanical overload (67) (Figure 4b).

Conceptually, the difference between cell response and autoregulation is important. Autoregulation requires (*a*) closed-loop feedback, (*b*) sufficient feedback gain, and (*c*) interaction with the environment (i.e., external load, ECM, neighboring cells). These elements are present in cardiomyocytes: (*a*) MCT provides closed-loop feedback in the dynamic E-C coupling system, (*b*) the feedback gain is tuned by MCT-Ca^2+^ gain, and (*c*) the cell–ECM and cell–cell connections transmit mechanical strain and stress in the myocardium. The autoregulation of E-C coupling in cardiomyocytes was first predicted in a conceptual model (66), which guided experimental research and discovery (67). In turn, the experimental data provided realistic measures for incorporating MCT-Ca^2+^ gain into the next mathematical model (72). To understand autoregulation of the dynamic E-C coupling system, it is essential to combine experiments and modeling in future research.

A paradigm shift to an autoregulatory E-C coupling system in cardiomyocytes opens a promising line of research into the cellular and molecular mechanisms underlying the heart’s intrinsic autoregulation in response to load changes in health and diseases.

### 4.2. Arrhythmogenic Activities in the Cardiomyocyte Under Mechanical Overload

Experiments from several groups consistently showed that mechanical loading led to spontaneous Ca^2+^ sparks and Ca^2+^ waves in mouse or rat cardiomyocytes (69, 70, 78). Interestingly, rabbit cardiomyocytes did not display Ca^2+^ sparks but showed discordant alternans in the Ca^2+^ transient amplitude and the action potential duration (82). This species difference might be because the SR-Ca^2+^ content in cardiomyocytes is high in rodents but low in larger mammal hearts (i.e., rabbit, human). Hence, stress-induced MCT might overload SR-Ca^2+^ to cause diastolic Ca^2+^ sparks and waves in rodents but only upregulates SR-Ca^2+^ incrementally to cause alternans in rabbit (and possibly in human). Both spontaneous Ca^2+^ waves and discordant alternans may lead to arrhythmogenic activities in the heart (140–142).

### 4.3. Open Issues and Perspectives

The heart has one major function: to adequately supply blood to the body. The heart must maintain cardiac output by continuously regulating its contraction in response to moment-to-moment changes in activity, posture, and emotions. It has been more than 100 years since the fundamental discoveries—associated with the names of Frank, Starling, and Anrep—were made on the heart’s intrinsic ability to adapt its contractile force in response to mechanical load changes. Here, we review progress in the field and propose new perspectives in understanding the autoregulation mechanisms intrinsic of the heart. Looking forward, we would like to highlight some open issues and hypotheses that warrant further research.

1. The adaptive response of the heart to an acute increase in outflow resistance is shown in Figure 1a. PV loop B shows that the initial response is strain-dominated. PV loop C shows that the long-term response is stress-dominated. Cardiomyocytes must generate approximately the same force in these two scenarios. How do the cardiomyocyte’s architecture, structural elements, mechanosensors, and MCT mechanisms work together to distinguish stress from strain?
2. Discordant results between labs might be due to the different techniques used to apply mechanical loads to cardiomyocytes (e.g., 1D stretch versus 3D viscoelastic matrix). Different experimental settings engage different mechanosensors that detect different local strain and stress (i.e., internal axial stress versus surface traction). There are many putative mechanosensors and MCT pathways. How do the plurality of mechanosensors and the network of MCT pathways interact? Are there critical molecular hubs that integrate MCT signaling? How do MCT pathways cross talk with other signaling pathways such as β-adrenergic signaling? Future research should seek to identify the mechanosensors and decipher their downstream MCT pathways.
3. We propose that the F-S mechanism and Anrep effect are not independent processes but are synergistic and dynamically linked at the cellular and molecular levels. If this proposition is correct, then a change in one will affect the other, which may have important implications on the impact of volume overload and pressure overload in the development of cardiac arrhythmias and heart failure.

### 4.4. A Mechano-Centric View on Cardiac Function and Heart Diseases

From a mechano-centric view, the heart functions as a smart pump that can adapt to changing mechanical load to maintain the output. To understand the heart’s autoregulation mechanism, it is critically important to decipher MCT pathways and how they feed back to regulate the E-C coupling dynamic system.

Mechanical overload is associated with various heart diseases. Dilated cardiomyopathy involves high ventricular wall stress because of wall thinning and chamber enlargement (143). The high wall stress demands that cardiomyocytes must contract more strongly in order to maintain stroke volume (144). Hypertrophic cardiomyopathy involves stiffened myocardium and impaired relaxation that cause diastolic dysfunction. Hypertensive heart disease is associated with chronic pressure overload on the ventricular wall. Intriguingly, these seemingly disparate diseases all involve high risks of cardiac arrhythmias (143, 144). Given that MCT regulates the E-C coupling dynamic system, it is plausible that MCT may help coordinate the E-C coupling dynamics in healthy hearts but lead to arrhythmogenesis in diseased hearts.

The MCT mechanisms enable a healthy heart to autoregulate contractility to maintain stroke volume and cardiac output despite physiological load changes. In heart failure, however, the diseased hearts can no longer adapt to changing load and fail to maintain adequate cardiac output. A therapeutic goal for treating heart failure is to increase the force generation to enhance cardiac output and restore the load-dependent adaptation. The clinical guidelines for the management of heart failure (145) recommend reducing preload and afterload using drugs (e.g., diuretics, ACE inhibitors, beta blockers). However, targeting the exact molecular mechanisms underlying various forms of heart failure are yet to be developed. If our proposed synergy between the F-S mechanism and Anrep effect is correct, then bolstering both will have a multiplicative effect on force generation. Many potential molecular targets in the MCT pathways might increase the F-S mechanism and Anrep effect. Innovative therapeutic strategies would bolster intrinsic MCT mechanisms that increase these effects.

Deciphering the MCT pathways and the key molecular players will also inform treatment of heart diseases caused by genetic defects in the MCT pathways. For example, DMD is caused by mutations in the dystrophin gene that lead to severe neuromuscular dysfunction and fatal arrhythmias and cardiomyopathy (146). Recently, the US Food and Drug Administration approved a gene therapy for increasing dystrophin expression in DMD patients (147). Knowing that the dystrophin glycoprotein complex is a prominent mechanosensor in cardiomyocytes, deciphering the MCT pathway downstream from dystrophin will help develop effective therapies.

Systemic diseases such as hypertension and diabetes can also lead to development of hypertrophy, arrhythmias, and heart failure. Studies show that the onset of hypertension immediately activates the MCT mechanisms to cause E-C coupling remodeling, and sustained chronic hypertension further leads to progressive structural remodeling with hypertrophic growth, arrhythmias, and heart failure (148). Better understanding of the MCT mechanisms underlying hypertension will help elucidate the stage-by-stage development of heart diseases, which will be essential for developing effective therapies for early-stage prevention and late-stage mitigation.

## Supplementary Material

Video S1_Strain

Video S2_Stress

Video S3_Surface Traction

## Footnotes

1 The change in the fractional change in sarcomere length is *ΔL/L* ~ Δ*EDV/EDV*, where Δ*EDV* is the change from the reference *EDV*. The dimensionless ratio, Δ*L/L*, is called the strain. This relationship stems from the fact that the ventricular volume scales as the cube of the characteristic length of the heart, taken to be the mean radius.
